# Gene and microRNA analysis of neutrophils from patients with polycythemia vera and essential thrombocytosis: down-regulation of micro RNA-1 and -133a

**DOI:** 10.1186/1479-5876-7-39

**Published:** 2009-06-04

**Authors:** Stefanie Slezak, Ping Jin, Lorraine Caruccio, Jiaqiang Ren, Michael Bennett, Nausheen Zia, Sharon Adams, Ena Wang, Joao Ascensao, Geraldine Schechter, David Stroncek

**Affiliations:** 1 Department of Transfusion Medicine, Clinical Center, National Institutes of Health, Bethesda, Maryland, USA; 2 Department of Hematology, Emek Hospital, Afula, Israel; 3 Hematology Section, Veterans Affairs Medical Center, Washington DC, USA

## Abstract

**Background:**

Since the V617F mutation in JAK2 may not be the initiating event in myeloprofilerative disorders (MPDs) we compared molecular changes in neutrophils from patients with polycythemia vera (PV) and essential thrombocythosis (ET), to neutrophils stimulated by G-CSF administration and to normal unstimulated neutrophils

**Methods:**

A gene expression oligonucleotide microarray with more than 35,000 probes and a microRNA (miR) expression array with 827 probes were used to assess neutrophils from 6 MPD patients; 4 with PV and 2 with ET, 5 healthy subjects and 6 healthy subjects given G-CSF. In addition, neutrophil antigen expression was analyzed by flow cytometry and 64 serum protein levels were analyzed by ELISA.

**Results:**

Gene expression profiles of neutrophils from the MPD patients were similar but distinct from those of healthy subjects, either unstimulated or G-CSF-mobilized. The differentially expressed genes in MPD neutrophils were more likely to be in pathways involved with inflammation while those of G-CSF-mobilized neutrophils were more likely to belong to metabolic pathways. In MPD neutrophils the expression of CCR1 was increased and that of several NF-κB pathway genes were decreased. MicroRNA miR-133a and miR-1 in MPD neutrophils were down-regulated the most. Levels of 11 serum proteins were increased in MPD patients including MMP-10, MMP-13, VCAM, P-selectin, PDGF-BB and a CCR1 ligand, MIP-1α.

**Conclusion:**

These studies showed differential expression of genes particularly involved in inflammatory pathways including the NF-κB pathway and down-regulation of miR-133a and miR-1. These two microRNAs have been previous associated with certain cancers as well as the regulation of hyperthrophy of cardiac and skeletal muscle cells. These changes may contribute to the clinical manifestations of the MPDs.

## Introduction

The chronic myeloproliferative disorders (MPDs) are clonal hematopoietic disorders that involve multiple cell lineages. They include polycythemia vera (PV), essential thrombocytosis (ET) and primary myelofibrosis (PMF) [1]. A mutation in the gene encoding Janus Kinase 2 (JAK2), which is involved with hematopoietic growth factor signaling, has been found in almost all patients with PV and about half those with ET [2-5]. This mutation, JAK2 V617F, is a gain of function mutation and hematopoietic progenitor cells from patients with this mutation have increased sensitivity to hematopoietic growth factors [5].

While JAK2 V617F has been found in neutrophils from many patients with chronic MPDs, it is not clear if JAK2 V617F is the initiating lesion in MPDs nor is the complete spectrum of the molecular changes associated with these disorders known. Germline JAK2 V617F mutations have not been found in familial MPD, however, somatic JAK2 V617F mutations have been identified in some affected kindreds [6,7]. Furthermore, first degree relatives of MPD patients have a 5- to 7-fold elevated risk of MPD, but the gene(s) or factors that predispose relatives to PV, ET and MF are not known [8]. This suggests that there are heritable alleles that predispose individuals to the acquisition of JAK2 V617F and the development of MPD [1,9]. Further characterization of the molecular changes in MPD neutrophils could lead to a better understanding of the development of these diseases and their clinical manifestations.

This study further characterized the molecular changes in neutrophils from patients with MPDs by comparing neutrophils from healthy subjects using global gene and microRNA (miR) expression arrays. The expression of neutrophil proteins was also assessed by flow cytometry and the levels of serum inflammatory factors by ELISA. Since G-CSF signals through JAK2 MPD neutrophils were also compared to those of healthy subjects after five days of G-CSF administration. In this way genes and miR could be identified whose change in expression was not due to constitutive activation by *JAK2 V617F*.

## Methods

### Study Design

These studies were approved by institutional review boards at the NIDDK, NIH and Veterans Administration Medical Center, Washington DC. Whole blood was collected into EDTA tubes from patients with MPD, healthy subjects, and healthy subjects given G-CSF. Neutrophils isolated from the EDTA blood was used for gene expression and microRNA analysis. For MPD patients whole blood was also collected into citrate tubes and was used to isolate neutrophils for JAK V617F analysis. Blood collected in tubes without anticoagulant was used to obtain serum for protein analysis. WHO criteria was used to make the diagnosis of PV and ET [10].

### G-CSF Mobilization of Granulocytes

Healthy subjects were given 10 micrograms/kg of G-CSF (filgrastim, Amgen, Thousand Oaks, California, USA) subcutaneously daily for 5 days. Blood was collected for analysis approximately 2 hours after the last dose of G-CSF was given.

### Neutrophil Isolation

Whole blood, 6 mL in EDTA (K2 EDTA 1.8 mg/mL, BD Vacutainer, Becton, Dickinson and Company, Franklin Lakes, NJ), was collected from healthy donors, MPD patients and donors following a course of G-CSF treatment. Percoll (Sigma, St. Louis, Missouri, USA) density gradients were used to isolate the neutrophils. Briefly, gradients were prepared by gently overlaying 63% Percoll solution on top of 72% Percoll solution, in equal volumes. Prior to overlaying the whole blood sample on the gradient, the majority of red blood cells were removed via sedimentation by diluting whole blood 1:2 with hetastarch (Hespan; 6% heta starch in 0.9% sodium chloride, B. Braun Medical Inc., Irvine, California, USA) and incubating for approximately 20 minutes at room temperature. After layering the leukocyte rich/heta starch solution on the gradient, the sample was centrifuged at 1,500 rpm for 25 minutes with no brake upon centrifuge deceleration. The neutrophil layer was harvested from the interface between the two Percoll solutions and washed twice with physiologic saline.

### Flow cytometry for Surface Markers

Flow cytometry analysis of granulocyte surface markers was performed on fresh whole blood samples. Cells were stained with monoclonal antibodies against CD177-FITC, CD15-FITC (Chemicon International, Temecula, CA), CD64-FITC, CD16-FITC, CD18-FITC, CD11b-FITC (Caltag Laboratories, Buckingham, UK) CD10-PE, CD31-PE, CD44-FITC, CD45-FITC, CD55-FITC, CD59-FITC, CD62L-FITC (eBiosciences, San Diego, CA) and incubated at 4°C for 30 minutes in the dark. Mouse IgG isotype controls were also used (Caltag Laboratories). The FACSCalibur flow cytometer and CellQuest Pro software (BD Biosciences, San Jose, CA) were used for analysis by acquiring 10,000 events and determining the viable neutrophil population by light scatter.

### Assessment of JAK2 V617F

Isolated neutrophils were tested for JAK2 V617F by DNA sequencing. V617F mutations were identified utilizing sequence-based typing methodology. Primary amplification of the specific region of JAK2 utilized primers *Jak2-1 (pf) *= **tgc tga aag tag gag aaa gtg cat **and *Jak2-2 (pr, sr) *= **tcc tac agt gtt ttc agt ttc aa **which produced a 345bp product. After primary amplification, sequence primers *Jak2-5 (sf) *= **agt ctt tct ttg aag cag caa **and *Jak2-2 (pr, sr) *= **tcc tac agt gtt ttc agt ttc aa **were utilized for detection of the V617F mutation. Conditions included the use of 2.0 mM Mg++, 3 pmole of primer, GeneAmp 10× PCR Gold Buffer, 0.35 unit of AmpliTaq gold DNA polymerase (ABI) 5 U/ul, and 0.15 mM each of 10 mM dNTP mixture (Amersham) with Big Dye Terminator^® ^Cycle Sequencing kits (Applied Biosystems). Template DNA was utilized at a concentration of 40–60 ug/mL. PCR cycling parameters were 95°C for 10 minutes; 95°C for 30 seconds → 52°C for 40 seconds → 72°C for 40 seconds = 40 cycles; 72°C for 2 minutes and hold at 4°C. Sequencing reactions were run on an Applied Biosystem 3730xL DNA Analyzer and analyzed utilizing standard alignment software.

### RNA Preparation, RNA Amplification and Labeling for Oligonucleotide Microarray

Total RNA from harvested neutrophils was extracted using Trizol reagent according to the manufacturer's instructions (Invitrogen, Carlsbad, California, USA). The quality of secondary amplified RNA was tested with the Agilent Bioanalyzer 2000 (Agilent Technologies, Waldbronn, Germany) and amplified into antisense RNA (aRNA) as previously described [11]. Also total RNA from peripheral blood mononuclear cells pooled from six normal donors was extracted and amplified into aRNA to serve as the reference. Pooled reference and test aRNA were isolated and amplified in identical conditions to avoid possible interexperimental biases. Both reference and test aRNA were directly labeled using ULS aRNA Fluorescent Labeling kit (Kreatech, Amsterdam, The Netherlands) with Cy3 for reference and Cy5 for test samples. Whole-genome human 36 K oligonucleotide arrays were printed in the Infectious Disease and Immunogenetics Section of the Department of Transfusion Medicine, Clinical Center, NIH (Bethesda, Maryland, USA) using oligonucleotides purchased from Operon (Operon, Huntsville, Alabama, USA). The Operon Human Genome Array-Ready Oligo Set version 4.0 contains 35,035 oligonucleotide probes, representing approximately 25,100 unique genes and 39,600 transcripts excluding control oligonucleotides. The design is based on the Ensembl Human Database build (NCBI-35c) with full coverage on NCBI human Refseq dataset (04/04/2005). The microarray is composed of 48 blocks and one spot is printed per probe per slide. Hybridization was carried out in a water bath at 42°C for 18 to 24 hours and the arrays were then washed and scanned on a GenePix 4000 scanner at variable photomultiplier tube to obtain optimized signal intensities with minimum (<1% spots) intensity saturation. The resulting data files were uploaded to the mAdb database  and further analyzed using BRBArrayTools developed by the Biometric Research Branch, National Cancer Institute .

### MicroRNAs Expression Profiling

A microRNA probe set was designed using mature antisense microRNA sequences (Sanger data base, version 9.1) consisting of 827 unique microRNAs from human, mouse, rat and virus plus two control probes. The probes were 5' amine modified and printed in duplicate on CodeLink activated slides (General Electric, GE Health, New Jersey, USA) via covalent bonding in the Immunogenetics Laboratory, DTM, CC, NIH. 4 μg total RNA isolated by using Trizol reagent (Invitrogen, Carlsbad, California) was directly labeled with miRCURY™ LNA Array Power Labeling Kit (Exiqon, Woburn, Massachusetts, USA) according to manufacture's procedure. The total RNA from an Epstein-Barr virus (EBV)-transformed lymphoblastoid cell line was used as the reference for the microRNA expression array assay. The test sample was labeled with Hy5 and the reference with Hy3. After labeling, the sample and the reference were co-hybridized to the microRNA array at room temperature overnight in the presence of blocking reagents as previously described [12] and the slides were washed and scanned by GenePix scanner Pro 4.0 (Axon, Sunnyvale, California, USA). Resulting data files were uploaded to the mAdb database  and further analyzed using BRBArrayTools developed by the Biometric Research Branch, National Cancer Institute .

### Array Data Processing

For analysis of the gene and microRNA array data, the raw data set was filtered according to a standard procedure to exclude spots with minimum intensity that was arbitrarily set to an intensity parameter of 200 for gene expression data and 100 for microRNA array data in both fluorescence channels. Spots flagged by the analysis software and spots with diameters <20 μm for gene expression array and <10 μm for the microRNA array were excluded from the analysis.

The filtered data were normalized using median over entire array and were retrieved by the BRB ArrayTool  developed at the National Cancer Institute (NCI), Biometric Research Branch, Division of Cancer Treatment and Diagnosis. Hierarchical cluster analysis was conducted on the genes or microRNA using Cluster and TreeView software [13]. For annotation of genes and functional pathways, the Database for Annotation, Visualization and Integrated Discovery (DAVID) 2007 software [14] and Ingenuity Pathway Analysis software  was used. All microRNA target prediction analysis used BRB ArrayTool microRNA targets program , TargetScan  and miRBase Targets .

### Gene and MicroRNA Expression Quantitative PCR

To validate the microarray analysis, 5 genes and 2 microRNAs were selected for Quantitative PCR. Gene expressions for TNFAIP3 (Assay ID, Hs00234713_m1), NFKBIE (Assay ID, Hs00234431_m1), NFKBIA (Assay ID Hs00153283_m1), CBS (Assay ID Hs00163925_m1) and MCL1(Assay ID Hs03043899_m1) were quantified by TaqMan Gene Expression Assays (Applied Biosystems, Foster City, California, USA) according to manufacturers' protocol and normalized by GAPDH (Assay ID Hs99999905_m1) PCR amplification of target genes and quantification of the amount of PCR products were performed by ABI PRISM 7900 HT Sequence Detection System (Applied Biosystems). Differences in expression were determined by the relative quantification method; the Ct values of the test genes were normalized to the Ct values of endogenous control GAPDH. The fold change was calculated using the equation 2^-ΔΔCt^.

Differentially expressed microRNAs, miR-133a (Assay ID, 4373142) and miR-219 (Assay ID, 4373080), were measured by TaqMan microRNA Assays (Applied Biosystems, Foster City, California, USA) as previously reported [15]. The differences of expression were determined by relative quantification method; the Ct values of microRNAs were normalized to the Ct values of endogenous control RNU48 (Assay ID 4373383). The fold change was calculated using the equation 2^-ΔΔCt^.

### Analysis of Serum Proteins

Serum samples were collected and frozen immediately, and stored at -80°C until further analysis. The serum samples were analyzed by protein expression profiling. The level of 64 soluble factors were assessed on an ELISA-based platform (Pierce Search Light Proteome Array, Boston, MA) consisting of multiplexed assays that measured up to 16 proteins per well in standard 96 well plates (Table 1). The 64 factors were selected to included hematopoietic factors, factors associated with inflammation, and those previously found to be increased in the serum of healthy subjects given G-CSF [16].

**Table 1:** Serum factors measured in MPD patients and healthy subjects

IL-1α	MCP-1 (CCL2)	TPO	TNFα
IL-1β	MCP-2 (CCL8)	G-CSF	INFα
IL-2	MCP-3 (CCL7)	GM-CSF	TGFα
IL-6	MCP-4 (CCL13)	MMP-1	PDGFAA
IL-10	E-Selectin	MMP-2	PDGFAB
IL-11	P-Selectin	MMP-8	PDGFBB
IL-2R	L-Selectin	MMP-9	HGF
IL-4R	MIP-1α (CCL3)	MMP-10	VCAM
IL-6R	MIP-1β (CCL4)	MMP-13	ICAM-1
TARC (CCL17)	MIP-1δ	TIMP-1	PECAM-1
OPN	MIP-3α (CCL20)	TIMP-2	FASL
IP-10	MIP-3β (CCL13)	MPO	CD40L
Eotaxin (CCL11)	MIG (CXCL9)	SAA	RANK
ITAC (CXCL11)	IP-10 (CXCL10)	SDF-1b (CXCL12)	RANKL
ENA-78 (CXCL5)	GROα (CXCL1)	OPG	RANTES (CCL5)
Exodus II	GROγ (CXCL3)	LIF	TNFR1

### Statistical Analysis

Unsupervised analysis was performed by using BRBArrayTools  and the Stanford Cluster Program [17]. Class comparison analysis was performed using parametric unpaired Student's t-test to identify differentially expressed genes or microRNA among different sample groups and using different significance cutoff levels as demanded by the statistical power of each comparison. Statistical significance and adjustments for multiple test comparisons were based on univariate and multivariate permutation tests as previously described [18,19].

## Results

### Global Transcriptome Analysis

**Figure 1:**
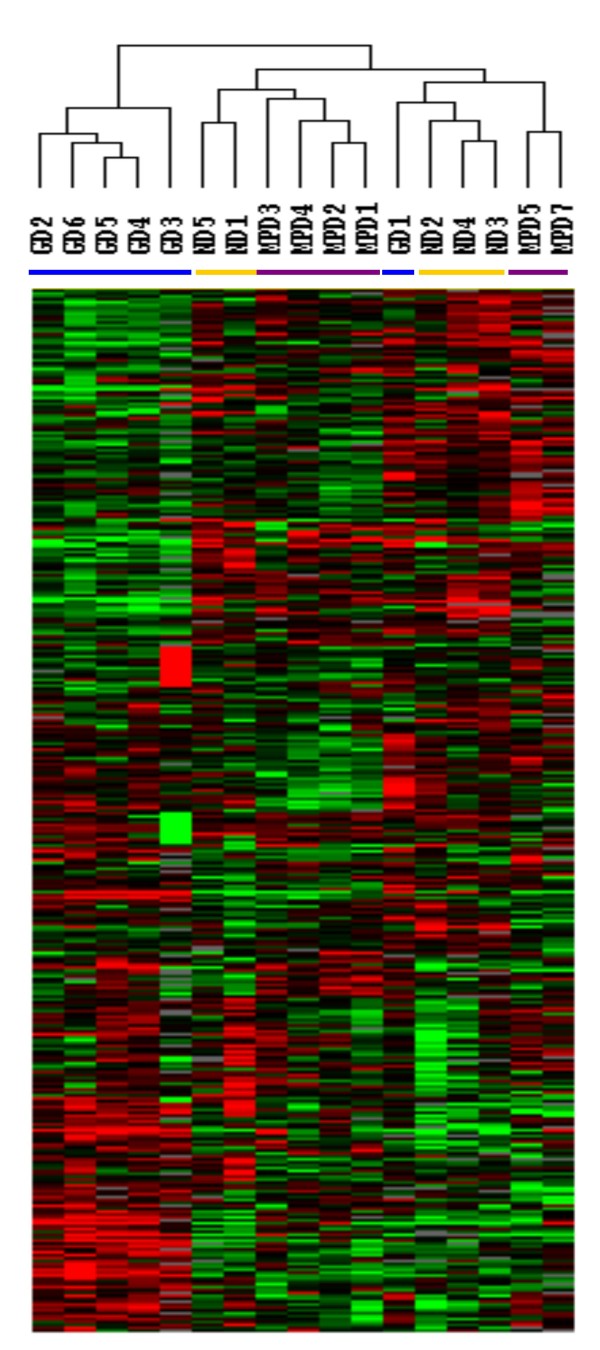
**Gene expression analysis of MPD neutrophils**. Gene expression of neutrophils from 6 MPD patients, 5 healthy subject neutrophils and 6 healthy subjects given G-CSF was analyzed using a microarray with more than 35,000 probes. The 3,617 genes that were expressed in at least 80% of samples and were up-regulated at least two-fold in one sample were analyzed by unsupervised hierarchical clustering of Eisen. The purple bar indicates neutrophils from patients with MPDs and the yellow bar those from healthy subjects and the blue bar from healthy subjects given G-CSF.

Neutrophils from 6 MPD patients were studied; 4 with PV and 2 with ET. JAK2 V617F was detected in 3 of the 4 PV patients and in 1 of the 2 ET patients (Table 2). Global gene expression analyses of neutrophils from 6 subjects with MPDs were compared with 6 healthy subjects given 5 days of G-CSF and the 5 healthy subjects. Among the 17 samples and 35,000 probes in the array, 3,617 were expressed by 80% of the samples and their expression was increased by 2-fold or greater in at least one sample. Unsupervised hierarchical clustering analysis of these 3,617 genes revealed three distinct groups: the G-CSF group which included 5 of the 6 G-CSF mobilized neutrophil samples, the MPD group with 4 of the 6 MPD neutrophil samples and 2 healthy subject neutrophils, and the mixed group with 3 healthy subject, 2 MPD, and 1 G-CSF-mobilized neutrophils (Figure 1).

**Table 2:** Gender, race, age, diagnosis and JAK2 V617F status of patients whose neutrophils were analyzed for gene and microRNA expression profiling

**Patient**	**Gender**	**Race**	**Age (years)**	**Diagnosis**	**JAK2 V617F**
1	Female	Caucasian	45	ET	Positive
2	Male	Caucasian	47	ET	Negative
3	Female	Caucasian	63	PV	Positive
4	Male	Caucasian	62	PV	Positive
5	Female	Caucasian	57	PV	Negative
7	Male	Caucasian	52	PV	Positive

ET = essential thrombocytosis

PV = polycythemia vera

**Figure 2:**
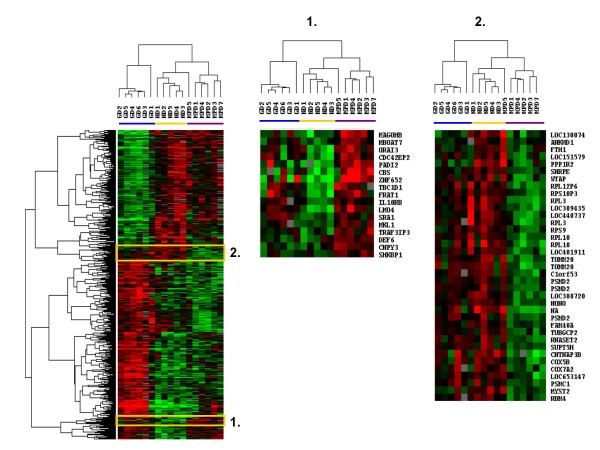
**Gene expression profiling of differentially expressed MPD neutrophil genes**. The 1,006 genes differentially expressed among 6 MPD patients, 5 healthy subjects and 6 subjects given 5 days of G-CSF (F-test, p < 0.005) were analyzed by hierarchical clustering of Eisen. Genes in cluster 1 were up-regulated only in MPD neutrophils and those in cluster 2 were down-regulated only in MPD neutrophils. The purple bar indicates neutrophils from patients with MPDs and the yellow bar those from healthy subjects and the blue bar from healthy subjects given G-CSF.

These results showed that the gene expression profile of MPD neutrophils differed from that of healthy subject neutrophils and G-CSF-mobilized neutrophils. Further analysis found that the expression of 1,006 genes differed among neutrophils from the MPD patients, healthy subjects, and healthy subjects given G-CSF (F-test, p ≤ 0.005). Hierarchical clustering analysis of these 1,006 genes separated the neutrophils into 3 groups; one contained neutrophils from 5 of 6 MPD patients, another included neutrophils from 5 healthy subjects and 1 MPD patient, and the third contained neutrophils from all 6 subjects given G-CSF (Figure 2). In this gene expression profile the MPD neutrophils aligned closer to the healthy subject neutrophils than the G-CSF-mobilized neutrophils. Two clusters of genes distinguished the MPD neutrophils from the healthy subject neutrophils. One cluster was made up of 17 genes whose expression was increased more in MPD neutrophils than in neutrophils from healthy subjects or healthy subjects given G-CSF (Figure 2, cluster 1) and another contained 38 genes down-regulated in MPD neutrophils but not in healthy subjects or G-CSF mobilized neutrophils (Figure 2, cluster 2). The cluster of MPD up-regulated genes included FRAT1, ZNF652, LMO4, IL10RB, and cystathionine β-synthase (CBS). FRAT1 is a regulator of the Wnt signaling pathway and is overexpressed in esophageal squamous cell carcinoma [20]. ZNF652 has a role in the suppression of breast oncogenesis and vulvar cancer [21,22]. LMO4 is a transcription regulator and increased expression of LMO4 in pancreatic ductal adenocarcinoma is associated with a survival advantage [23]. The expression of CBS has been previously reported to be up-regulated in neutrophils from patients with MPDs [24]. Among the down-regulated genes were ribosomal proteins including 3 copies of RPL10, 2 copies of RPL3, and RPS9, RPS10P3, and RPL12P6; proteosome proteins including 3 copies of PSMD2 and PSMC; and cytochrome c oxidases COX5B and COX7A2.

**Figure 3:**
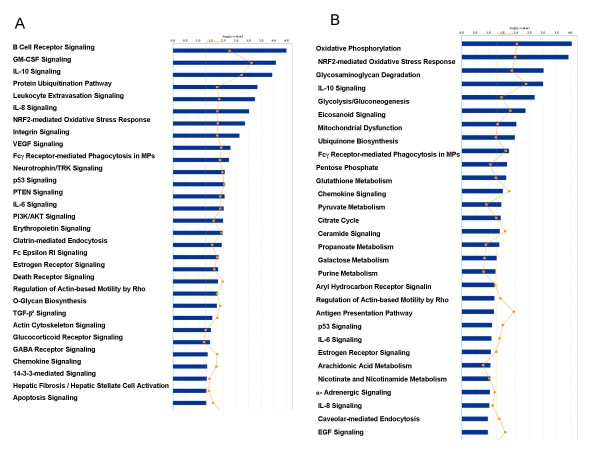
**Panel A. Pathway analysis of differentially expressed MPD genes**. Ingenuity pathway analysis showing canonical pathways significantly modulated by the genes whose expression differed among the MPD neutrophils compared to healthy subject neutrophils(p < 0.05). A total of 1,270 genes were differentially expressed: 473 were up-regulated and 800 were down-regulated. Only the 30 pathways with the most significant changes are shown. The p value for each pathway is indicated by the bar and is expressed as -1 times the log of the p value. The line represents the ratio of the number of genes in a given pathway that meet the cutoff criteria divided by the total number of genes that make up that pathway. Panel B. Pathway analysis of differentially expressed G-CSF genes. Ingenuity pathway analysis showing canonical pathways significantly modulated by the genes whose expression differed among the G-CSF-mobilized neutrophils compared to healthy subject neutrophils (p < 0.05). A total of 909 genes were differentially expressed: 452 were up-regulated and 457 were down-regulated. Only the 30 pathways with the most significant changes are shown. The p value for each pathway is indicated by the bar and is expressed as -1 times the log of the p value. The line represents the ratio of the number of genes in a given pathway that meet the cutoff criteria divided by the total number of genes that make up that pathway.

To further explore the differences between MPD and G-CSF-mobilized neutrophils, the genes differentially expressed in MPD neutrophils compared to healthy subject neutrophils were identified as well as those differentially expressed in G-CSF-mobilized-neutrophils. MPD neutrophil differentially expressed genes were more likely to belong to inflammatory pathways (Figure 3A). In contrast, G-CSF-mobilized neutrophils differentially expressed genes were more likely to belong to metabolic pathways (Figure 3B).

To further characterize MPD neutrophils, we identified those differentially expressed genes whose expression was increased or decreased to the greatest fold as compared to the healthy subjects. Among the 30 genes whose expression was increased to the greatest extent in MPD neutrophils were ZNF652, CBS, LMO4, AXUD1, MCL1 and CCR1 (Table 3). AXUD1 is a regulator of the Wnt signaling pathway and is down-regulated in lung, kidney, and colon cancer [25]. MCL-1 is a member of the Bcl-2 family and is an important anti-apoptotic molecule for multiple types of hematopoietic cells [26]. CCR1 is a chemokine receptor for at least 11 different chemokines including CCL3 (MIP-1α), CCL5 (RANTES), CCL7 (MCP-3), CCL8 (MCP-2), CCL14, CCL15, CCL16 and CCL23 [27]. Among the genes down-regulated most in MPD neutrophils were neutrophil elastase 2 (ELA2) and two NF-kβ pathway genes (NFKBIA and NFKBIE) all of which are involved in inflammation (Table 4).

**Table 3:** Genes up-regulated the most in MPD neutrophils compared to those from healthy subjects (p < 0.05, tests)

**Gene**		**Fold increase**	**p**
Rg9mtd1	PREDICTED: RNA (guanine-9-) methyltransferase domain containing 1 (Rg9mtd1)	4.79	0.00844

HPR	haptoglobin-related protein (HPR)	4.55	0.000443

*ZDHHC19*	*zinc finger, DHHC-type containing 19 (ZDHHC19)*	*4.34*	*0.00278*

ZNF652	zinc finger protein 652 (ZNF652)	3.90	5.90E-05

ADCY3	adenylate cyclase 3 (ADCY3)	3.64	0.0121

PROK2	Prokineticin 2	3.60	0.000166

C19orf59	chromosome 19 open reading frame 59 (C19orf59)	3.33	0.0139

ZFYVE21	zinc finger, FYVE domain containing 21 (ZFYVE21)	3.32	0.00362

CCR1	chemokine (C-C motif) receptor 1 (CCR1)	3.14	0.000335

EGR1	early growth response 1 (EGR1)	3.13	0.0229

ST3GAL4	ST3 beta-galactoside alpha-2,3-sialyltransferase 4 (ST3GAL4)	3.13	0.00225

PADI2	peptidyl arginine deiminase, type II (PADI2)	3.12	2.90E-06

AXUD1	AXIN1 up-regulated 1 (AXUD1)	3.08	0.00546

LOC728488	PREDICTED: similar to Nuclear envelope pore membrane protein POM 121 (Pore membrane protein of 121 kDa) (P145) (LOC728488)	3.06	0.00241

	Transcribed locus, moderately similar to XP_001235777.1 PREDICTED: hypothetical protein [Gallus gallus]	3.04	0.0123

CBS	cystathionine-beta-synthase (CBS)	2.97	0.00183

	CDNA: FLJ21549 fis, clone COL06253	2.96	0.00649

ACRV1	acrosomal vesicle protein 1 (ACRV1), transcript variant 11.	2.91	0.00574

UPF2	UPF2 regulator of nonsense transcripts homolog (yeast)	2.84	0.0179

GYG1	glycogenin 1 (GYG1)	2.75	0.0146

NTRK2	neurotrophic tyrosine kinase, receptor, type 2 (NTRK2), transcript variant c	2.73	0.00792

LMO4	LIM domain only 4 (LMO4)	2.69	0.000128

MCL1	myeloid cell leukemia sequence 1 (BCL2-related) (MCL1), transcript variant 1	2.67	0.000287

LOC729915	PREDICTED: similar to Nuclear envelope pore membrane protein POM 121 (Pore membrane protein of 121 kDa) (P145) (LOC729915)	2.57	0.0172

GALNT14	UDP-N-acetyl-alpha-D-galactosamine:polypeptide N-acetylgalactosaminyltransferase 14 (GalNAc-T14) (GALNT14)	2.57	0.00853

FAM69A	family with sequence similarity 69, member A (FAM69A)	2.57	0.0446

MED26	Mediator complex subunit 26	2.56	0.0109

C1orf115	chromosome 1 open reading frame 115 (C1orf115)	2.55	0.0309

KIFC3	kinesin family member C3 (KIFC3)	2.54	0.00290

Rg9mtd1	Transcribed locus	2.53	0.0113

**Table 4:** Genes down-regulated the most in MPD neutrophils compared to those from healthy subjects (p < 0.05, t-tests)

**Gene**		**Fold Increase**	**p**
TPMT	thiopurine S-methyltransferase (TPMT)	6.90	2.14 × 10^-4^

	CDNA FLJ35883 fis, clone TESTI2008929	4.47	0.00636

ZNF75	zinc finger protein 75 (D8C6) (ZNF75), mRNA.	4.29	3.24 × 10^-3^

FAM3B	family with sequence similarity 3, member B (FAM3B), transcript variant 2	4.20	2.11 × 10^-3^

UBE2D4	ubiquitin-conjugating enzyme E2D 4 (putative) (UBE2D4)	4.10	3.44 × 10^-3^

AK2P2	PREDICTED: adenylate kinase 2 pseudogene 2 (AK2P2)	3.63	8.43 × 10^-3^

XP_933530.1	PREDICTED: hypothetical protein XP_933530 [Source:RefSeq_peptide_predicted;Acc:XP_933530]	3.61	6.61 × 10^-4^

PVRL2	poliovirus receptor-related 2 (herpesvirus entry mediator B) (PVRL2), transcript variant alpha	3.27	0.0418

	CDNA FLJ38039 fis, clone CTONG2013934	3.13	9.00 × 10^-7^

NFKBIA	nuclear factor of kappa light polypeptide gene enhancer in B-cells inhibitor, alpha (NFKBIA)	3.11	4.23 × 10^-3^

NFKBIA	nuclear factor of kappa light polypeptide gene enhancer in B-cells inhibitor, alpha (NFKBIA)	3.07	5.02 × 10^-3^

GADD45B	growth arrest and DNA-damage-inducible, beta (GADD45B), mRNA.	3.06	0.0158

PER1	period homolog 1 (Drosophila) (PER1), mRNA.	2.92	6.84 × 10^-3^

C9orf89	chromosome 9 open reading frame 89 (C9orf89), mRNA.	2.91	3.51 × 10^-4^

DYNC1LI1	dynein, cytoplasmic 1, light intermediate chain 1 (DYNC1LI1)	2.89	2.53 × 10^-3^

RYBP	RING1 and YY1 binding protein (RYBP)	2.88	7.27 × 10^-3^

WRB	tryptophan rich basic protein (WRB)	2.85	2.21 × 10^-3^

ELA2	elastase 2, neutrophil (ELA2)	2.82	0.0180

CNTNAP3B	OTTHUMP00000046146|hypothetical protein LOC389722|novel protein similar to contactin associated protein-like 3 (CNTNAP3)	2.82	6.20 × 10^-6^

UBE2E2	ubiquitin-conjugating enzyme E2E 2 (UBC4/5 homolog, yeast) (UBE2E2)	2.80	8.14 × 10^-4^

ARL10	ADP-ribosylation factor-like 10 (ARL10)	2.79	6.80 × 10^-3^

RPS28	ribosomal protein S28 (RPS28)	2.76	1.28 × 10^-4^

C15orf29	chromosome 15 open reading frame 29 (C15orf29)	2.76	9.34 × 10^-3^

C20orf199	chromosome 20 open reading frame 199 (C20orf199)	2.71	2.28 × 10^-5^

GADD45B	Growth arrest and DNA-damage-inducible, beta	2.69	5.09 × 10^-3^

NFKBIE	nuclear factor of kappa light polypeptide gene enhancer in B-cells inhibitor, epsilon (NFKBIE)	2.66	0.0271

SCARB1	scavenger receptor class B, member 1 (SCARB1), transcript variant 1	2.63	0.0485

TSP50	testes-specific protease 50 (TSP50)	2.62	8.76 × 10^-3^

EFR3B	PREDICTED: EFR3 homolog B (S. cerevisiae) (EFR3B)	2.60	0.021

MLSTD1	male sterility domain containing 1 (MLSTD1)	2.59	0.0134

**Figure 4:**
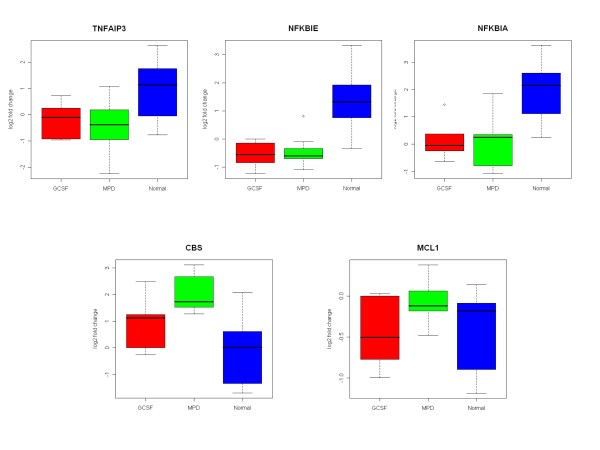
**Analysis of differentially expressed MPD neutrophil genes by quantitative real time PCR (RT-PCR)**. The expression of five genes NFKBIA, NFKBIE, TNFAIP3, MCL1 and CBS in MPD neutrophils was analyzed by qRT-PCR. The expression of NFKBIA, NFKBIE, and TNFAIP3 were down-regulated in MPD and G-CSF-mobilized neutrophils. The expression of CBS was significantly increased in MPD neutrophils. The expression of MCL1 was also increased in MPD neutrophils but the difference was not significant. The results of analysis by qRT-PCR and gene expression profiling were similar.

We used qRT-PCR to further confirm the differential expression of 3 NFKB pathway genes, NFKBIA, NFKBIE and TNFAIP3 as well as MCL1 and CBS (Figure 4). This confirmed that the expression of NFKBIA, NFKBIE, and TNFAIP3 were significantly down-regulated in both MPD and G-CSF-mobilized neutrophils compared to those from healthy subjects. The expression of CBS was significantly up-regulated in MPD neutrophils and the expression of MCL1 was up-regulated but not to a significant degree as compared to healthy subjects.

### Micro RNA Expression Results

MicroRNA expression was compared among MPD, G-CSF-mobilized and healthy subject neutrophils using a microarray. Among the 827 probes, 500 remained after selecting only those expressed in >80% of samples. Unsupervised hierarchical clustering analysis of the neutrophil samples separated the samples into two groups. One group included 3 G-CSF-mobilized neutrophils and 3 healthy subject neutrophils and the second included 3 G-CSF-mobilized neutrophils, 6 MPD neutrophils and 5 normal donor neutrophils (data not shown).

**Figure 5:**
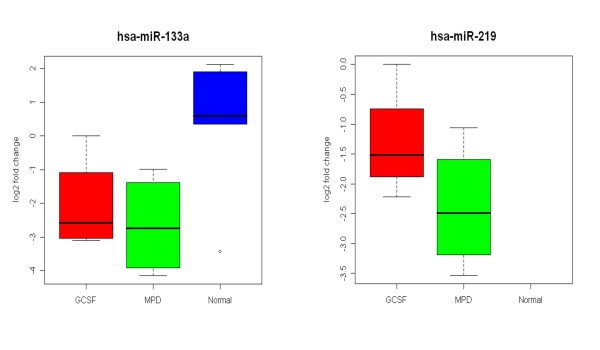
**Analysis of differentially expressed MPD neutrophil microRNA by quantitative real time PCR (qRT-PCR)**. The expression of miR-133a and miR-219 were analyzed by qRT-PCR. The expression of miR-133a was down-regulated in both MPD and G-CSF-mobilized neutrophils while that of miR-219 was up-regulated in MPD and G-CSF-mobilized neutrophils. In fact, no miR-219 transcripts were detected in neutrophils from healthy subjects. The results of analysis by qRT-PCR and microRNA expression profiling were similar.

Comparison of the expression of microRNA between MPD and healthy subject neutrophils found that the expression of 21 microRNA were up-regulated in MPD neutrophils and 11 were down-regulated (p < 0.05). Among the microRNA up-regulated in MPD neutrophils were 5 that were increased more than 2-fold; miR-219, miR-515-5p, miR-142-5p, miR-143, and miR-101 (Table 5). The up-regulation of miR-219 in MPD neutrophils compared to those from healthy subjects was confirmed by qRT-PCR (Figure 5). Interestingly, miR-219 has been found to be expressed in the brain and its levels exhibit circadian rhythms and are involved in the control of the suprachiasmatic nuclei (SCN), the master circadian clock in mammals [28]. The expression of 142–5p has also been found to be increased in peripheral blood leukocytes [12]. MicroRNA miR-143 has been found to be involved with cell differentiation. The differentiation of pre-adipocytes to adipocytes is associated with the increased levels of miR-143 [29]. Bruchova and colleagues have found that miR-143 is up-regulated in neutrophils from patients with polycythemia vera [30]. The expression of miR-143 is down-regulated in B cell malignancies, Burkitt's lymphoma cell lines [31], and colorectal cancer [32].

**Table 5:** MPD neutrophil differentially expressed microRNA (miR)*

**Up-regulated miR**		**Down-regulated miR**	
**Description**	**Fold change**	**Description**	**Fold change**

hsa-miR-219	4.11	hsa-miR-133a	3.41
hsa-miR-515-5p	2.63	hsa-miR-504	2.73
hsa-miR-142-5p	2.47	hsa-mir-565	2.52
hsa-miR-143	2.43	hsa-miR-1	2.16
hsa-miR-101	2.21	hsa-miR-216	2.14
hsa-miR-424	1.93	hsa-miR-485-5p	1.76
hsa-miR-450	1.92	hsa-miR-483	1.71
hsa-miR-301	1.86	hsa-mir-657	1.62
hsa-miR-33	1.86	hsa-miR-502	1.59
hsa-miR-19b	1.81	hsa-mir-615	1.43
hsa-miR-29b	1.76	hsa-mir-421	1.32
hsa-miR-30a-5p	1.73		
hsa-miR-29c	1.70		
hsa-miR-185	1.66		
hsa-miR-21	1.63		
hsa-miR-19a	1.6		
hsa-miR-200b	1.48		
hsa-miR-542-3p	1.43		
hsa-mir-625	1.42		
hsa-miR-106b	1.33		
hsa-miR-20b	1.31		

* p < 0.05 compared to healthy subject neutrophils

Among the microRNA down-regulated in MPD neutrophils the expression of five were decreased more than 2-fold: miR-133a, miR-504, miR-565, miR-1, and miR-216 (Table 5). The down-regulation of miR-133a in MPD neutrophils was confirmed by qRT-PCR (Figure 5). MicroRNA miR-133a and -1 are clustered on the same chromosome and are transcribed together as a single transcript [33,34]. These two microRNA are preferentially expressed in brown adipocytes [35], cardiac, and skeletal muscle [34] and are important in the differentiation and regulation of cardiac and skeletal muscle. Little is known about miR-216, -504 and -565. Micro RNA-216 is expressed by the pancreas. A comparison of normal pancreas with 33 other tissues found that the expression of miR-216 and miR-217 and the lack of expression of miR-133a were characteristic of pancreatic tissue [36].

### Serum Protein Levels

**Figure 6:**
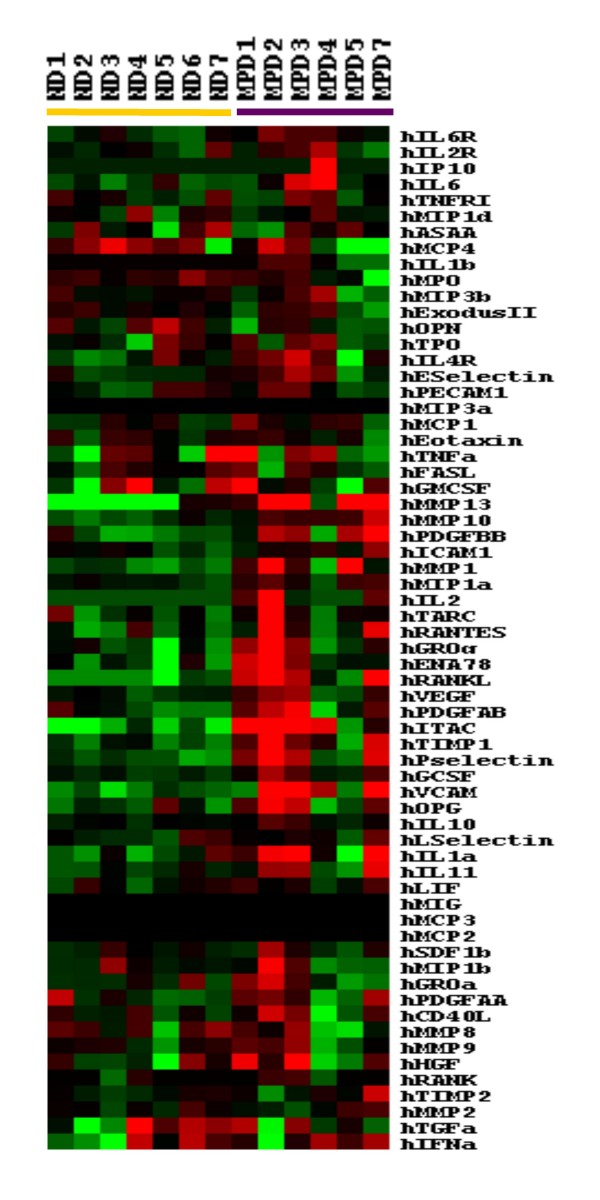
**Comparison of serum protein levels among MPD patients and healthy subjects**. Levels of each of the 64 factors were measured by nested ELISA in 6 MPD patients and 7 healthy subjects and the levels were analyzed by supervised hierarchical clustering of Eisen. Higher factor levels were indicated in red and lower levels in green. Samples from MPD patients are shown by the purple bar and from healthy subjects by the yellow bar.

The levels of 64 serum proteins were compared in the 6 MPD patients and 7 healthy subjects. The levels of the 64 factors in each of the 6 MPD patients and 7 healthy controls were analyzed by supervised hierarchical clustering analysis (Figure 6). The MPD samples were characterized by 33 proteins whose levels were greater than in healthy subjects. Eleven of these were significantly increased in MPD patients compared to healthy subjects (t-tests, p < 0.05, Table 6) and included 2 chemokines (CXCL11 and CCL3), a cytokine (IL-1a), 2 matrix metalloproteinases (MMPs) (MMP-10 and MMP-13), growth factors (PDGF-BB and G-CSF) VCAM, TIMP-1, IL-6R and P-selectin.

**Table 6:** Serum factors whose levels differed between MPD patients and healthy subjects.

**Factor**	**Healthy Subjects (n = 7)**	**MPD Patients** **(n = 6)**	**P**
VCAM	1,707,211 ± 5,080	10,467,524 ± 7,793,493	0.0123
MMP-10	716 ± 195	1,672 ± 854	0.0145
MIP-1α (CCL3)	62.6 ± 9.9	93.5 ± 27.8	0.0185
MMP-13	54.1 ± 63.1	1,181 ± 1091	0.0190
IL-6R	5,215 ± 1,606	8,421 ± 2,684	0.0220
TIMP-1	287,485 ± 89,954	930,916 ± 650,021	0.0209
P selectin	131,558 ± 35,298	527,593 ± 45,1417	0.0249
ITAC (CXCL11)	21.0 ± 13.0	338 ± 330	0.0263
G-CSF	61.1 ± 7.5	109.0 ± 52.9	0.0352
PDGFBB	473.1 ± 239	1,962 ± 1,665	0.0381
IL-1α	11.1 ± 6.5	39.4 ± 32.1	0.0421

Values are expressed as mean ± SD in pg/ml

### Expression of Neutrophil Membrane Molecules

Neutrophil expression of CD11b, CD15, CD16, CD18 and CD177 was analyzed by flow cytometry in 24 patients with MPD (11 PV and 13 ET). JAK2 V617F was detected in 13 of the 24 patients and one was homozygous (Table 7). Expression was compared to 43 healthy subjects and 27 healthy subjects who were given 5 daily doses of G-CSF.

**Table 7:** Comparison of neutrophil expression of CD11b, CD15, CD16, CD18, and CD177 among MPD patients, healthy subjects, and healthy subjects given G-CSF

	**Healthy Subjects** **(n = 43)**	**All MPD Patients** **(n = 24)**	**Polycythemia Vera** **(n = 11)**	**Essential Thrombocytosis** **(n = 13)**	**G-CSF-Treated Subjects** **(n = 27)**
	
**% Reactive cells**					
CD11b	55 ± 26	54 ± 28	66 ± 27	44 ± 26†	64 ± 24
CD15	21 ± 25	50 ± 31*†	51 ± 31*†	49 ± 33*†	23 ± 29
CD16	81 ± 22	82 ± 19	83 ± 24	82 ± 16	89 ± 5
CD18	48 ± 33	73 ± 26*	73 ± 30*	73 ± 23*	62 ± 35
CD177	53 ± 23	59 ± 28†	59 ± 29†	58 ± 27†	82 ± 26*

**Mean Fluorescence Intensity**					

CD11b	182 ± 51	187 ± 107	171 ± 100	200 ± 115	155 ± 67
CD15	480 ± 284	374 ± 236	373 ± 265	377 ± 221	441 ± 443
CD16	2,946 ± 1,345	2,580 ± 1,138†	2,410 ± 1,430†	2,725 ± 853†	890 ± 336*
CD18	451 ± 300	250 ± 81*	267 ± 100	237 ± 61*	253 ± 107*
CD177	625 ± 383	575 ± 267†	587 ± 251†	566 ± 290†	2,012 ± 1088*

* p < 0.05 compared to healthy subjects

† p < 0.05 compared to subjects given G-CSF

Fluor = fluorescence

**Figure 7:**
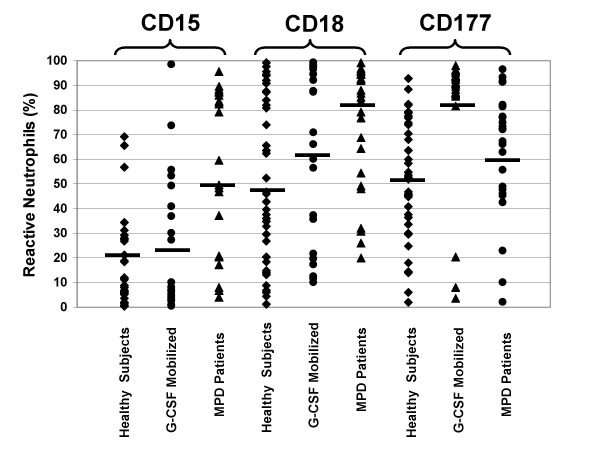
**Comparison of the expression of CD15, CD18, and CD177 by neutrophils from MPD patients, healthy subjects, and healthy subjects given G-CSF**. Neutrophil expression of CD15, CD18, and CD177 was analyzed by flow cytometry in 24 MPD patients and 43 healthy subjects. The results are expressed as a percent of neutrophils that were reactive with each antibody. The expression of CD15 and CD18 was significantly greater in MPD neutrophils compared to those from healthy subjects, but there was no difference in the expression of CD15 and CD18 between neutrophils from healthy subjects given G-CSF and those who were not. The expression of CD177 was increased in G-CSF-mobilized neutrophils compared to unmobilized healthy subject and MPD neutrophils, but there was no difference in CD177 expression between MPD and unmobilized healthy subject neutrophils.

CD15 and CD18 expression differed among MPD patients and healthy subjects, but not that of CD11b, CD16 or CD177. More neutrophils expressed CD15, Lewis-x, in people with MPD than in healthy subjects (50 ± 31% versus 21 ± 25%, p < 0.0002) (Table 7, Figure 7). This was the case for both subjects with PV and ET. The proportion of neutrophils expressing CD18 was also increased in people with MPD (73 ± 26% versus 48 ± 33%, p < 0.003), although the mean neutrophil fluorescent intensity was reduced (250 ± 81 versus 451 ± 300, p < 0.003) (Table 7, Figure 7), but was similar to G-CSF stimulated neutrophils. Both the proportion of neutrophils expressing CD177 and the mean fluorescence intensity of neutrophils were increased slightly in MPD neutrophils, but these changes were not significant.

Following G-CSF administration, the expression of CD16 and CD18 as assessed by the mean fluorescence intensity decreased (Table 7, Figure 7). In contrast, the number of neutrophils expressing CD177 and the mean fluorescence intensity of CD177 expression increased.

The expression of several other neutrophil adhesion molecules, Fc receptors and other antigens were compared in the same cohort of 6 MPD patients in whom gene and miR expression profiles and serum proteins were measured; 4 with PV and 2 with ET. The proportion of neutrophils expressing CD64 was greater in MPD patients than in healthy subjects (13 ± 9% versus 6 ± 4%, p < 0.05) but not the mean fluorescence intensity (373 ± 73 versus 201 ± 63). There was no difference in the expression of CD10, CD31, CD44, CD45, CD55, CD59, and CD62L among neutrophils from MPD patients and healthy subjects (data not shown).

## Discussion

In order to better characterize the molecular basis of MPDs, we compared gene and miRNA expression profiles of neutrophils from MPD patients with those from healthy subjects. We identified several genes and microRNA whose expression differed in MPD neutrophils compared to those of healthy subjects. Since most patients with PV and approximately half with ET have a gain-of-function mutation in JAK2, we also compared MPD neutrophils with neutrophils from healthy subjects treated with G-CSF, a hematopoietic growth factor that signals through JAK2. While there were similarities in gene expression signatures in MPD neutrophils and G-CSF-mobilized neutrophils, we also found several differences. The expression of a greater number of genes was changed in G-CSF-mobilized neutrophils compared to MPD neutrophils. There were also a number of genes whose expression changed in MPD neutrophils, but not in G-CSF-mobilized neutrophils. In addition, several microRNAs were differentially expressed by MPD neutrophils. Many of these gene and microRNA expression changes were similar to those found in hypertrophied cells, cancers, and hematologic malignancies.

Among the microRNA that were down-regulated in MPD neutrophils were two closely associated down-regulated microRNA; miR-133a and miR-1. These two miR are located in the same bicistronic unit on chromosome 18, are transcribed together [34], and are involved in skeletal muscle and myocardial muscle differentiation and proliferation. The down-regulation of miR-133a and miR-1 is associated with hypertrophic myocardium and skeletal muscle [33,37-39]. The suppression of miR-133 has been shown to induce cardiac hypertrophy [37]. miR-133a down-regulation has been noted in squamous cell carcinoma of the tongue [40,41]. In addition, the expression of miR-1 is also reduced in heptocellular carcinoma [42] and lung cancer [43]. Down-regulation of these two microRNAs may play a role in the proliferation of hematopoietic cells in MPDs.

Gene expression analysis found that MPD neutrophils exhibited a pro-inflammation profile. MPD differentially expressed genes included those involved with B cell, IL-6, IL-8, VEGF, TGF-β, Fcε RI and integrin signaling pathways. These changes are not simply due to the constitutive activation of JAK2 since they were not present in G-CSF-mobilized neutrophils. Instead, most G-CSF-mobilized neutrophils differentially expressed genes were in metabolic and synthesis pathways.

Analysis of specific genes whose expression changed in MPD neutrophils identified several genes in the NF-κB pathway. Change in expression of 3 of these genes was confirmed by qRT-PCR. The expression of several NF-κB genes were increased and several were decreased so the overall effect on the pathway is not certain, however, the NF-κB pathway is likely important in MPD. NF-κB promotes the survival, proliferation, differentiation and survival of lymphocytes and plasma cells [44,45]. NF-κB is also activated in chronic myeloid leukemia (CML) [46], but it has not been reported to be activated in MPDs [44]. In CML increased levels of NF-κB may be a down stream effect of brc-abl activation [46]. In our studies we also found that the expression of many NF-κB pathway genes were changed in neutrophils by G-CSF and it may be that constitutive activation of JAK2 in MPD results in NF-κB activation in PV and ET neutrophils.

The expression of CCR1 was increased in MPD patients. CCR1 is an important leukocyte chemokine receptor for several ligands including CCL3 or MIP-1α. The levels of 11 serum factors were elevated in ET and PV patients including CCL3 which can be a chemoattractant to activated neutrophils. These results suggest that the increased expression of CCR1 and CCL3 may contribute to the pro-inflammatory profile of MPD neutrophils.

Changes in serum protein levels and neutrophil antigen expression in PV and ET patients do not appear to be simply a result of constitutive activation of neutrophil JAK2. G-CSF signals through JAK2, but changes in these markers are different in healthy subjects given G-CSF than those in MPD patients. The levels of several factors are elevated in subjects given G-CSF that were not elevated in MPD patients including E-selectin, L-selectin, MMP-1, MMP-8, IL-2R, IL-10, IL-2R, TNFR1, hepatocyte growth factor (HGF) and SAA [16]. In addition several serum factors were changed in MPD patients that were not changed in healthy subjects given G-CSF including CXCL11, CCL3, PDGFBB, IL-1a, TIMP1, and P-selectin [16]. Changes in the levels of these serum proteins may be due to shedding or internal cellular sequestration of their receptors in hematopoietic cells, an inability of the receptor to bind the factor normally, or to increased protein production.

The elevation of many of these proteins could contribute to the clinical manifestations of ET and PV. Changes in serum and plasma protein levels have been studied in patients with PMF which is characterized by bone marrow myelofibrosis, extramedullary hematopoiesis and the presence of immature myeloid cells in the peripheral blood [47]. The release of proteolytic enzymes by PMF mononuclear cells is thought to contribute to the abnormal trafficking of CD34+ cells in PMF patients by degrading HPC adhesion molecules expressed on bone marrow stromal cells and thereby releasing hematopoietic progenitor cells (HPCs) into the circulation. The levels of soluble proteases MMP-9 and neutrophil elastase and VCAM-1 are increased in PMF patients [48]. MMP-9 and elastase are thought to cleave VCAM-1 expressed by stromal cells which leads to the disruption of the interaction of VCAM-1 and very late antigen -4 (VLA-4) expressed by HPCs resuling in the release of HPCs. The levels of peripheral blood CD34+ cells are also increased in PV patients and proteases likely contribute to the mobilization of HPCs in PV patients. We found that VCAM-1 levels were also increased in MPD patients as well as the levels of the proteolytic enzymes MMP-13 and MMP-10. The levels of MMP-9 and MMP-2 were also greater in MPD patients, but the difference was not significant.

Other factors may also contribute to the increased levels of circulating HPCs in MPD patients. G-CSF is an important mobilizer of HPCs and CD34+ cells. We found that G-CSF levels were increased in MPD patients. The levels of CCL3, a chemokine that can mobilize HPCs, were also increased in the MPD patients. Elevated levels of both G-CSF and CCL3 may contribute to HPC mobilization in MPD patients.

We also compared the expression of neutrophil surface proteins in ET and PV patients and healthy subjects, but found few differences. Neutrophil expression of CD18 and CD15 was up-regulated in MPD patients. Others have found that the expression of CD18 and CD11b was up-regulated on MPD neutrophils [49,50]. CD15 functions as a neutrophil adhesion molecule [51] and it is expressed by some types of leukemic cells [52] and by Reed-Sternberg cells [53] but its expression has not been previously analyzed on MPD neutrophils. We confirmed using a larger sample size the findings of Klippel and colleagues that the expression of CD177 is not increased although *CD177 *mRNA levels are markedly elevated in MPD neutrophils [54].

Comparison of MPD and G-CSF-mobilized neutrophil gene and antigen expression suggests that the changes in MPD neutrophils differ from those induced by G-CSF. These differences may be due to MPD-associated changes in other cell types. While G-CSF primarily affects neutrophils and neutrophil precursors, JAK2 V617F is found in neutrophils, neutrophil precursors, megakaryoctyes and red cell precursors. It may be that the constitutive activation of JAK2 in megakaryocytes and/or red cell precursors results in the secretion of factors by these cells that affects neutrophils.

JAK2 V617F is an important biomarker for MPD, but it would be useful to identify additional new MPD biomarkers. While the levels of 11 serum factors were elevated in ET and PV patients including VCAM-1, MMP-13, CXCL11, IL-1a, TIMP-1, PDGF-BB and P-selectin whose levels were more than 3-fold greater than the levels in healthy subjects, it is not likely that any of these factors can be used alone as a biomarker for MPD since none was elevated in all MPD patients. The measurement of a combination of factors might serve as a useful biomarker for PV or ET, however, most of the elevated factors are important inflammatory factors and they are likely to be elevated in other disorders. Larger studies are needed which compare the levels of these factors among patients with PV and ET, healthy subjects, and subjects with other hematologic and inflammatory diseases to determine if unique combinations of changes in soluble factor levels are characteristic of these disorders.

## Conclusion

This study provides new sights into the molecular changes in ET and PV. PV and ET neutrophils were characterized by the down-regulation of miR-1 and miR-133a and changes in the expression of many genes involved in inflammation including those in the NF-κB pathway.

## Competing interests

The authors declare that they have no competing interests.

## Authors' contributions

SS designed the study, performed research, analyzed data and wrote the paper; PJ designed the study, performed research, analyzed data and wrote the paper; LC designed the study, preformed research, analyzed data and wrote the paper; JR designed the study, preformed research, and analyzed data; MB designed the study, analyzed the data and wrote the paper; NZ preformed research and analyzed the data; SA preformed research and analyzed the data; EW designed the study and wrote the paper; JA designed the study and wrote the paper; GS designed the research and wrote the paper; and DS designed the study, analyzed data and wrote the paper.
